# HIPER-CHAD: Hybrid Integrated Prediction-Error Reconstruction-Based Anomaly Detection for Multivariate Indoor Environmental Time-Series Data

**DOI:** 10.3390/s26010171

**Published:** 2025-12-26

**Authors:** Vandha Pradwiyasma Widartha, Chang Soo Kim

**Affiliations:** Department of Information System, Pukyong National University, Busan 608737, Republic of Korea; vandhapw@pukyong.ac.kr

**Keywords:** hybrid model, LSTM, VAE, anomaly detection, multivariate, time series

## Abstract

This study introduces the Hybrid Integrated Prediction-Error Reconstruction-based Anomaly Detection (HIPER-CHAD) model, which addresses the challenge of reliably detecting subtle anomalies in noisy multivariate indoor environmental time-series data. The main objective is to separate temporal modeling of normal behavior from probabilistic modeling of prediction uncertainty, ensuring that the anomaly score becomes robust to stochastic fluctuations while remaining sensitive to truly abnormal events. The HIPER-CHAD architecture first employs a Long Short-Term Memory (LSTM) network to forecast the next time step’s sensor readings, subsequently forming a residual error vector that captures deviations from the expected temporal pattern. A Variational Autoencoder (VAE) is then trained on these residual vectors rather than on the raw sensor data to learn the distribution of normal prediction errors and quantify their probabilistic unicity. The final anomaly score integrates the VAE’s reconstruction error with its Kullback–Leibler (KL) divergence, yielding a statistically grounded measure that jointly reflects the magnitude and distributional abnormality of the residual. The proposed model is evaluated on a real-world multivariate indoor environmental dataset and compared against eight traditional machine learning and deep learning baselines using a synthetic ground truth generated by a 99th percentile-based criterion. HIPER-CHAD achieves an F1-score of 0.8571, outperforming the next best model, the LSTM Autoencoder (F1 = 0.8095), while maintaining perfect recall. Furthermore, a time-step sensitivity analysis demonstrates that a 20-step window yields an optimal F1-score of 0.884, indicating that the proposed residual-based hybrid design provides a reliable and accurate framework for anomaly detection in complex multivariate time-series data.

## 1. Introduction

Identifying anomalies in time-series data is a vital task in a range of sectors, including financial fraud detection, network security, healthcare, and industrial monitoring [1]. The timely and accurate identification of anomalous patterns data points or subsequences that deviate significantly from the expected normal behavior is essential for maintaining system integrity, preventing catastrophic failures, and enabling proactive decision-making [2]. In indoor environmental monitoring and wireless sensor networks, sensor anomalies may stem from environmental factors (e.g., dust, volatile organic compounds, sudden temperature or humidity shifts) and hardware issues (e.g., aging, calibration drift, electronic noise) [3]. Poor sensor placement, configuration errors, and suboptimal measurement practices can also result in inaccurate readings that are unrelated to actual physical changes [4]. With the increasing number of sensors and the high-dimensional time-series data they generate, the challenge of detecting anomalies effectively is becoming more complex, requiring the development of sophisticated and robust methodologies [5].

Historically, anomaly detection relied on statistical methods, such as the 3-Sigma Rule, and traditional machine learning techniques, including One-Class Support Vector Machines (OC-SVM) and Isolation Forest (iForest) [6]. While these methods offer interpretability and computational efficiency, they frequently encounter challenges in accurately representing the complex temporal dependencies and non-linear relationships that characterize contemporary time-series data [7]. The limitations of these traditional approaches become particularly pronounced in complex, real-world systems where anomalies are rare, subtle, and context-dependent [8]. The emergence of deep learning has revolutionized the field, offering powerful tools to automatically learn complex feature representations from raw time-series data [9]. Reconstruction-based models, particularly Autoencoders (AEs) and Variational Autoencoders (VAEs), have emerged as a dominant paradigm [10]. These models are constructed to reduce the reconstruction error of typical data, based on the assumption that atypical data will produce a markedly higher reconstruction error [11]. The success of this approach is further enhanced by incorporating temporal-aware architectures, such as Long Short-Term Memory (LSTM) networks and Temporal Convolutional Networks (TCNs), to develop models such as the LSTM Autoencoder (LSTM-AE) and TCN Autoencoder (TCN-AE) [12,13,14]. Temporal deep learning models have exhibited exceptional efficacy in modeling sequential data, thereby enhancing the precision of anomaly detection [15].

Despite the significant progress in deep learning-based anomaly detection, several critical challenges persist, particularly when dealing with real-world, stochastic time-series data. First, while temporal models like LSTM-AEs offer improvements, there is frequently a trade-off between capturing long-term dependencies and maintaining computational efficiency [16]. Deterministic models face significant challenges in differentiating true anomalies from the stochastic uncertainty or noise that is characteristic of complex systems, which often results in a high incidence of false alarms. The severity of false alarms varies by application domain and can have serious consequences. In fire detection networks, false alarms waste emergency resources and public funds [17]. In intrusion detection systems, they undermine trust in security infrastructure. In medical wireless sensor networks (WSNs), specifically in ICU environments, approximately 80–99% of clinical alarms are false or clinically insignificant; this contributes to ‘alarm fatigue,’ which can potentially lead to the overlooking of actual emergencies [18]. Similarly, in IIoT systems, false alarms reduce operational efficiency and increase maintenance costs [19]. Thus, reducing false alarms while maintaining detection sensitivity represents one of the most persistent challenges in modern anomaly detection.

Additionally, determining an appropriate anomaly threshold, typically based on the reconstruction error distribution, is a complex task that directly influences the system’s precision and recall outcomes [20]. Most fundamentally, the effectiveness of anomaly detection models is significantly influenced by how the Ground Truth is defined [21]. Recent studies have shown that using flawed or inconsistent benchmarks can create a false sense of advancement, complicating the accurate comparison of models [22]. The accuracy of a model can vary widely depending on whether the ground truth is established through statistical methods (e.g., 99th Percentile), another model (such as iForest), or expert labeling [23]. To address the challenge of stochasticity and improve the robustness of anomaly scoring, we introduce the Hybrid Integrated Prediction-Error Reconstruction-based Anomaly Detection (HIPER-CHAD) framework. This method initially utilizes a Long Short-Term Memory (LSTM) network to conduct sequence prediction and determine the residual error between the forecasted and actual values. Subsequently, a Variational Autoencoder (VAE) is employed to model the distribution of this residual error, thereby effectively differentiating between normal stochastic fluctuations and genuine anomalous deviations. The significance of this proposed framework includes the following contributions:

- HIPER-CHAD employs a dual-stage LSTM–VAE architecture specifically designed to learn the residual error distribution, where the LSTM models normal temporal dynamics and the VAE operates on the resulting prediction residuals. This design advances anomaly detection by providing a statistically grounded anomaly score that is more robust to the inherent variability present in multivariate sensor data.
- By modeling the residual errors with a VAE, the framework explicitly quantifies the uncertainty associated with the predictions: anomalies are identified not only by large reconstruction errors, but by residuals that fall outside the learned probabilistic range of normal stochastic variation. This probabilistic treatment of residuals enables the use of more stable, data-driven anomaly thresholds and substantially reduces false positives caused by noise and small fluctuations in otherwise normal data.

## 2. Materials and Methods

### 2.1. Dataset and Data Collection

The experimental data utilized in this study is a real-world multivariate time-series dataset of indoor environmental parameters. The dataset was collected through the development and installation of custom low-cost sensors, subsequently placed in a laboratory environment for experiment purposes. The measurements comprising five distinct features: Carbon Dioxide (${\mathrm{CO}}_{2}$), Volatile Organic Compounds (VOC), Temperature, Humidity, and Dust Concentration. This dataset has high temporal resolution, with a recording interval of one minute. The dataset as a whole covers a period of approximately 124 days, starting on 8 March 2024 and ending on 9 July 2024, consisting of 178,560 data points.

### 2.2. Data Quality and Preprocessing

The integrity and suitability of the data for time-series analysis, a multi-step preprocessing pipeline was implemented to addressing data quality issues and standardizing the feature space. The following procedures were implemented as part of the research methodology:

1. Error-code handling and imputation, sensor readings with the exact value 9999.0 are identified as non-physical error codes which commonly used as a placeholder for sensor errors or malfunctions. A total of 18 instances of this value were identified across the features and removed them from the time series data. Each removed entry is replaced by the median of the corresponding feature over the full dataset [24].
  (1) $${\hat{x}}_{i,j}=\left\{\begin{array}{cc} \mathrm{Med}(X_{j}) & \mathrm{if}\;x_{i,j}=9999.0\\ x_{i,j} & \mathrm{otherwise} \end{array}\right.$$
2. Temporal Resampling and Aggregation Creating a uniform time base for sequential modeling and to reduce computational load, the data was resampled from its original minute-level resolution to a specific time interval. This resampling was performed using mean aggregation, where the average of all measurements within each time window interval was calculated. This step ensures a consistent temporal structure for the subsequent time-series models.
  (2) $${\bar{x}}_{k,j}=\frac{1}{|W_{k}|}\sum_{i\in W_{k}}{\hat{x}}_{i,j}$$
3. Feature Normalization Ensuring that all environmental parameters contributed equally to the model training, regardless of their inherent scale or unit of measurement. The RobustScaler from scikit-learn python library was applied, which transforms the data by subtracting the median and scaling the data to the interquartile range (IQR). This method is preferred over standard scaling (Z-score normalization) because it is less susceptible to the influence of remaining outliers, thereby providing a more stable and reliable feature representation for the anomaly detection models [25].
  (3) $$x_{k,j}^{\prime }=\frac{{\bar{x}}_{k,j}-Q_{2}\left({\bar{X}}_{j}\right)}{Q_{3}\left({\bar{X}}_{j}\right)-Q_{1}\left({\bar{X}}_{j}\right)}$$

### 2.3. Ground Truth Generation

In the context of unsupervised anomaly detection, a labeled ground truth is typically unavailable. To facilitate the comparative evaluation of the proposed and baseline models, a synthetic ground truth ($y_{\mathrm{true}}$) was generated based on the statistical properties of the test dataset. This approach allows for the calculation of standard classification metrics (e.g., F1-Score, Precision, Recall) necessary for objective model comparison. The synthetic ground truth was established using the 99th Percentile Threshold Method, which assumes that the most extreme data points in the dataset represent the true anomalies. This method involves two primary steps: calculating an initial anomaly score for every data point and then applying a percentile-based threshold to generate the binary labels. For each multivariate time-series sample $x_{i}$ in the test set, an initial statistical anomaly score, $S_{i}$, was calculated. This score quantifies the maximum deviation of any feature in the sample from its respective feature’s median, $\mathrm{Med}(X_{j})$, across the entire dataset.

Let $x_{i}=\left[x_{i,1},x_{i,2},\ldots ,x_{i,D}\right]$ be the *i*-th sample with *D* features. The anomaly score $S_{i}$ is mathematically formulated as:

(4) $$S_{i}=\max_{j=1}^{D}\left(|x_{i,j}-\mathrm{Med}\left(X_{j}\right)|\right)$$

This score $S_{i}$ represents the largest absolute difference between a feature value in the sample and the typical (median) value for that feature. Once the anomaly scores $S=S_{1},S_{2},\ldots ,S_{N}$ for all *N* samples are computed, the threshold τ is determined by the *P*-th percentile of the score distribution. In this study, the 99th percentile was selected, meaning P=99. The threshold τ is defined as:

(5) $$\tau ={\mathrm{Percentile}}_{99}\left(S\right)$$

The binary ground truth label $y_{\mathrm{true},i}$ for the *i*-th sample is then generated by comparing its anomaly score $S_{i}$ against the threshold τ: $y_{\mathrm{true},i}=\left\{\begin{array}{cc} 1 & \mathrm{if}\;S_{i}>\tau \;\left(\mathrm{Anomaly}\right)\\ 0 & \mathrm{if}\;S_{i}\leq \tau \;\left(\mathrm{Normal}\right) \end{array}\right.$.

Before adopting the 99th percentile thresholding strategy, we conducted preliminary experiments with several alternative approaches to generate ground-truth anomaly labels. First, we applied the three-sigma rule [26], assuming that observations falling outside three standard deviations from the mean are anomalous. We then evaluated an unsupervised model-based detector, Isolation Forest [27], to obtain anomaly scores and labels directly from the multivariate sensor data. In addition, we explored a simple weighted-voting scheme that combined the binary decisions from these detectors [28]. The 99th percentile of the HIPER-CHAD anomaly score yielded a more stable and interpretable separation between normal and anomalous behavior, and therefore was adopted as the primary ground-truth generation method in this study. The selection of the 99th percentile as the anomaly threshold is a common and statistically justified practice in unsupervised anomaly detection benchmarking [29]. Anomalies are, by definition, rare events. In numerous real-world datasets, the proportion of genuine anomalies is frequently less than 1% [30]. By establishing the threshold at the 99th percentile, the method effectively identifies the top 1% of the most statistically extreme observations as anomalies, thereby aligning with the anticipated low prevalence of anomalous events.

### 2.4. The HIPER-CHAD Model Architecture

The HIPER-CHAD model is designed to combine the strengths of temporal modeling and probabilistic modeling to enhance anomaly detection. The framework adopts a two-stage hybrid architecture designed to isolate and model the stochastic nature of prediction errors. It combines the temporal modeling power of a LSTM network with the probabilistic generative capabilities of a VAE [31]. This design is fundamentally rooted in the principle of residual analysis, which posits that anomalies are best detected not in the raw data itself, but in the unpredictable component of the data after a robust model has accounted for the normal temporal patterns [32].

**Figure 1:**
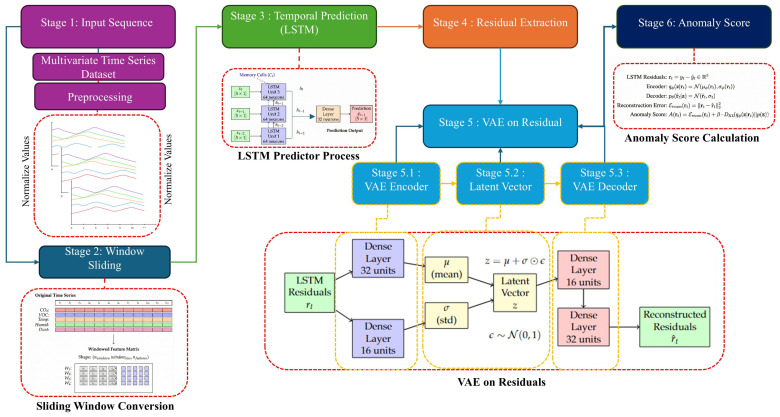
Architecture of HIPER-CHAD Framework model.

The architecture workflow that is shown in the Figure 1 demonstrates how to recognize various anomaly patterns that are not only individual in nature on a single sensor, but also collective and contextual patterns among many sensors, even under conditions of complex time correlations. The initial phase of the model workflow involves a data preparation phase (stages 1 and 2) transforms raw sensor inputs into a format suitable for temporal learning. In stage 1, a multivariate time-series dataset $X\in R^{T\times D}$, where *T* denotes timestamps and *D* represents sensor features (e.g., $CO_{2}$, temperature and etc). Preprocessing steps such as normalization and missing value imputation are applied to ensure numerical stability as explained in Section 2.2. Stage 2 introduces a sliding-window mechanism to capture temporal dependencies, converting the preprocessed data into sequences of *k* historical observations for subsequent prediction as defined in the formula below:

(6) $$X_{t-k+1:t}=\left\{x_{t-k+1},x_{t-k+2},\ldots ,x_{t}\right\}$$

The windowed data is subsequently provided as input to the LSTM predictor, which models temporal dependencies and generates next-step forecasts. These predictions are compared against the actual observations to compute residuals (prediction errors) at each time step. The resulting residuals serve as input to the residual-based VAE, a generative model that maps them into a low-dimensional latent space and reconstructs the residual vectors. At this stage, two principal components are employed to derive the anomaly score

1. The $L_{2}$ norm is frequently employed to quantify the reconstruction error between the original residual and the output reconstructed by the VAE, and
2. The Kullback–Leibler Divergence (KL divergence) between the latent distribution $q_{\phi }\left(z|x\right)$ and the standard normal prior p(z).

The total anomaly score for each time window is formulated as a combination of the reconstruction error and the KL divergence, with an adjustment parameter (typically denoted as β). These two components of the score are, respectively, the effective reconstruction error for distinguishing normal inputs that are familiar to the model, while the effective KL divergence captures residual patterns whose distribution deviates from the learned latent structure. By employing this hybrid scoring method, the model not only exhibits sensitivity to anomalies, which are characterized by extreme values, but also adeptly detects shifts in the residual distribution pattern that might not be immediately evident in the reconstruction phase.

#### 2.4.1. Temporal Prediction (LSTM Predictor) and Residual Extraction

Stages 3 and 4 constitute the core of temporal modeling, which is implemented using an LSTM-based sequence-to-point predictor. The primary goal is to capture the normal temporal dynamics of multivariate time-series data. The predictor takes as input a sequence of length of timewindow−1 and outputs the forecast for the value at the timewindow position. The architecture consists of:

- Input Layer: Takes sequences of shape (timewindow−1,Features).
- LSTM Layer: A single LSTM layer comprising 64 units and utilizing a Rectified Linear Unit (ReLU) activation function.
- Output Layer: A Dense layer comprising Features units and employing a linear activation function is utilized to predict the values of the subsequent time step.

The predictor is trained on the normal training data to minimize the Mean Squared Error (MSE) between the predicted and actual values. Normal training data is obtained by processing the entire sensor data history through sensor value cleaning by handling extreme outliers and replacing them with median values, resampling, and feature normalization using the robust scaler method. This stage produces a multivariate time series that is assumed to be dominated by normal behavior with a very small proportion of anomalies. This series is then converted into window sequences and divided chronologically, with the first 80% as training data and the last 20% as test data, all windows in the initial period are treated as normal without manual labeling, based on the assumption that the actual operation history contains few anomalies so that normal patterns remain dominant [33]. The LSTM predictor is trained using input from the training window except for the last time step as the target, learning normal temporal dynamics, then the training residual is calculated as the difference between the actual value and the LSTM prediction at the last time step, which is used as training input for the VAE so that the VAE specifically learns the normal prediction error distribution. The output of this stage is the residual error vector $r_{t}$, calculated as the difference between the actual observation $x_{t}$ and the predicted observation ${\hat{x}}_{t}$:

(7) $$r_{t}=x_{t}-{\hat{x}}_{t}$$

For normal data, this residual vector is expected to follow a predictable, low-magnitude distribution. Anomalies, however, are expected to result in a residual vector that deviates significantly from this learned distribution.

#### 2.4.2. Probabilistic Residual Model (VAE on Residuals)

The extracted residual vectors ($r_{t}$) are processed by a Variational Autoencoder (VAE) to model their probabilistic distribution. Unlike the deterministic LSTM predictor, this stage focuses on learning the stochastic properties of the prediction errors to differentiate between normal noise and genuine anomalies. The process is divided into three sub-stages as follows:

- Encoder: The encoder network functions to map the input residuals into a latent probabilistic representation. The input residuals are processed through a 32-unit dense layer to estimate the mean vector of the latent distribution. Simultaneously, the residuals pass through a 16-unit dense layer to determine the standard deviation (log-variance). The two parallel Dense layers produce the mean ($z_{\mathrm{mean}}$) and log-variance ($z_{\log\text{\_}\mathrm{var}}$) of the latent space.
- Latent vector: Using the learned parameters (μ and σ), the model generates a latent vector *z* through the reparameterization trick, enabling backpropagation during training. The sampling process is expressed as:
  (8) z=μ+σ⊙ϵ,ϵ∼N(0,1)
- Decoder: The decoder network aims to reconstruct the original residual vector from the sampled latent vector *z*. It comprises a Dense layer with 32 units utilizing a ReLU activation function, followed by a final Dense layer employing a linear activation function to reconstruct the input residual vector.

The VAE is trained with a custom loss function that balances the reconstruction loss (MSE) and the Kullback–Leibler (KL) divergence loss, with a weighting factor (KL_WEIGHT=0.01) to ensure the latent space is well-regularized and follows a standard normal distribution.

#### 2.4.3. Anomaly Scoring

The final HIPER-CHAD anomaly score (${\mathrm{Score}}_{\mathrm{HC}}$) for a test sample is a composite score derived from the VAE on the residual vector $r_{t}$:

(9) $${\mathrm{Score}}_{\mathrm{HC}}=\mathrm{MSE}\left(r_{t},{\hat{r}}_{t}\right)+\mathrm{KL}\text{\_}\mathrm{Divergence}\left(z_{\mathrm{mean}},z_{\log\text{\_}\mathrm{var}}\right)$$

This score effectively encapsulates two dimensions of anomaly detection: the magnitude of the reconstruction error, which indicates the VAE’s deficiency in accurately reconstructing the residual, and the probabilistic, which evaluates the degree to which the latent representation of the residual diverges from the established normal distribution. In practice, ${\mathrm{Score}}_{\mathrm{HC}}$ is a continuous quantity that is converted into a binary anomaly label by applying a threshold. Consistent with the ground-truth generation procedure described in Section 2.3, we adopt the 99th percentile of ${\mathrm{Score}}_{\mathrm{HC}}$ on the test set as the decision threshold: samples with ${\mathrm{Score}}_{\mathrm{HC}}$ above this percentile are labeled as anomalous, while the remaining samples are regarded as normal.

### 2.5. Hyperparameter Setting

In the proposed HIPER-CHAD model, hyperparameters were systematically selected to ensure reproducibility and architectural stability. The temporal modeling component utilizes an LSTM predictor configured with 64 hidden units, processing a lookback window of 20 time steps (k=20) to capture relevant trends. For the probabilistic modeling, the VAE architecture employs a specialized encoder with asymmetric dual dense layers—32 units for the mean vector (μ) and 16 units for the standard deviation (σ)—projecting inputs into a compact latent dimension (*z*) of 5. The decoder mirrors this complexity through sequential dense layers of 16 and 32 units. Training is performed using the Adam optimizer with a batch size of 32. A critical regularization parameter, the KL divergence weight (β), is set to 0.01 to prevent posterior collapse and ensure the model prioritizes accurate residual reconstruction.

## 3. Results

The comprehensive evaluation of the HIPER-CHAD model in comparison to eight other anomaly detection techniques provided clear evidence of its superior efficacy in identifying anomalies within multivariate indoor environmental time-series data. The experimental results, presented in Table 1, demonstrate a distinct hierarchy of performance among the nine evaluated anomaly detection models when assessed against the 99th Percentile Ground Truth. The baseline models utilize the fundamental architectures described in their respective citations. However, to ensure a fair comparison, we standardized the network capacity rather than using the disparate settings found in the original studies. The F1-Score served as the primary metric for comparison, offering a comprehensive evaluation of a model’s precision and recall.

**Figure 2:**
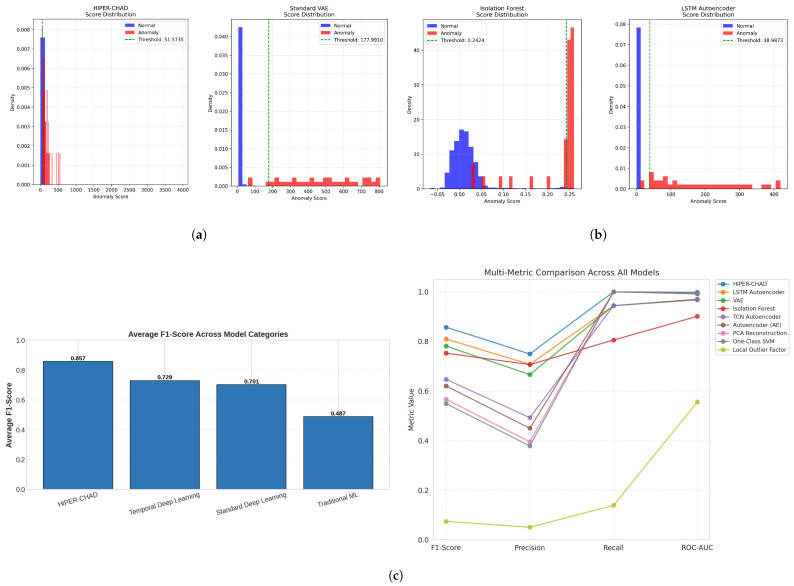
The comparative results across anomaly detection models. (**a**) The anomaly score distribution and threshold separation of proposed model (HIPER-CHAD) and VAE model from left to right respectively. (**b**) The anomaly score distribution and threshold separation of Isolation Forest and LSTM Autoencoder model from left to right respectively. this anomaly score showing how normal and anomalous samples are distinguished across different approaches. (**c**) Average F-score across temporal between proposed model, temporal deep learning, standard deep learning, and traditional ML (**left**), and comparative performance across evaluation for all models (**right**).

HIPER-CHAD achieved an F1-Score of 0.8571, reflecting a strong balance between accurately identifying true anomalies (Recall = 1.0000) and effectively minimizing false positives (Precision = 0.7500). HIPER-CHAD distinguished itself as the only model to attain a perfect recall rate of 100%, while also preserving high precision. The model achieved a ROC-AUC score of 0.9983, thereby affirming its exceptional proficiency in accurately ranking anomalies. The second-best model, the LSTM Autoencoder, achieved an F1-Score of 0.8095, representing a 5.9% relative improvement for HIPER-CHAD. The disparity in performance becomes even more evident when examining the average F1-Score across the model categories, as illustrated in Figure 2. HIPER-CHAD, the sole representative of the hybrid category, outperformed all other categories, affirming the effectiveness of the proposed two-stage method. The temporal models achieved an average F1-Score of 0.7286, which is 38.3% higher than the 0.5577 score of non-temporal models as shown in Figure 2c, underscoring the significance of temporal modeling for this data type. This quantitative advantage is further explained through a qualitative analysis of the distribution of anomaly scores of the four top models with the highest F1-score based on Table 1 shown in Figure 2a,b. As shown in the comparative histograms, traditional models like Isolation Forest exhibit significant distributional overlap between normal and anomalous samples, resulting in a higher rate of false alarms. In contrast, HIPER-CHAD showed well-separated classes. The proposed residual-based scoring mechanism ensures that normal data points are mapped to a highly compact distribution concentrated near zero, creating a clear and decisive margin from the anomalous events. This distinct bimodality facilitates the determination of a robust decision threshold, thereby minimizing classification ambiguity and directly explaining the model’s superior precision and recall balance compared to the more diffuse score distributions observed in baseline architectures.

### 3.1. Analysis of Reconstruction and Residual Errors

**Figure 3:**
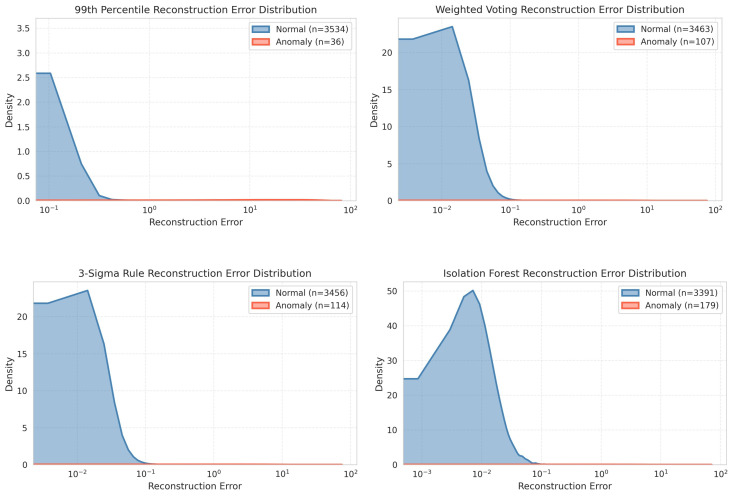
Reconstruction error distribution across ground truth anomaly labeling methods.

We conduct an analysis of both reconstruction errors and prediction residuals utilizing several ground truth methodologies. Reconstruction error is defined as the discrepancy between the actual sensor values and their reconstructed counterparts as generated by the model. Prediction residuals, which are derived from temporal models, denote the difference between forecasted and observed sensor data, thereby facilitating the identification of anomalous temporal variations or behaviors. This process is quantitatively and visually validated by the comparative results presented in Figure 3, where multiple ground truth strategies such as 3-Sigma Rule, 99th Percentile, Isolation Forest, and Weighted Voting are systematically benchmarked against ground truth. The error distribution plot shows a fairly clear difference in distribution: normal samples consistently show a very concentrated error density around zero, indicating the model’s ability to predict standard environmental dynamics. In contrast, anomalous events result in a flat, dispersed distribution extending into higher error magnitudes, as explicitly illustrated in the comparative density plots where the 99th Percentile ground truth exhibits the sharpest separation between the normal (blue) and anomalous (orange) classes compared to the more overlapping distributions observed in the Isolation Forest method. This observation suggests that percentile-based thresholds enforce stricter criteria for anomaly identification, thereby minimizing false positives at the cost of reduced anomaly coverage. Conversely, Isolation Forest demonstrates a broader anomaly detection capability, as reflected by its heavier tail and higher anomaly count, but introduces greater overlap with normal samples, which may compromise precision. The 3-Sigma rule and Weighted Voting approaches occupy intermediate positions, balancing sensitivity and specificity by maintaining concentrated normal distributions while allowing moderate anomaly dispersion. Among these methods, the 99th Percentile approach emerges as the most effective for this case, as it provides the clearest separation between normal and anomalous distributions, ensuring high precision and reducing misclassifications risk. This makes it particularly suitable for environments where false positives must be minimized to maintain operational reliability.

**Figure 4:**
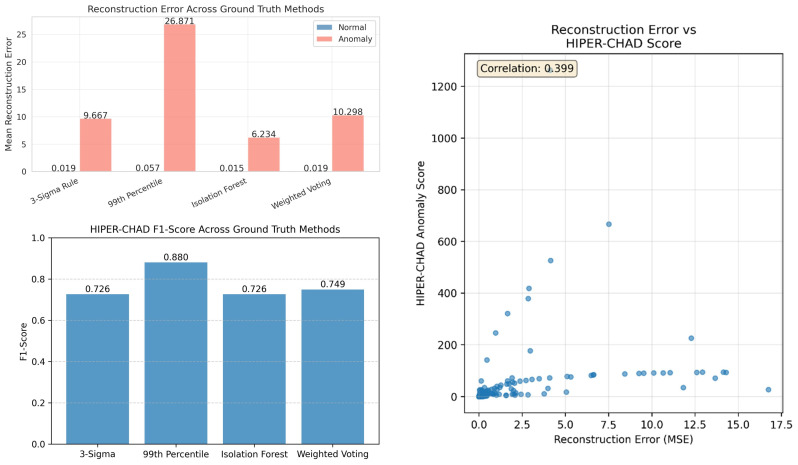
Performance evaluation of HIPER-CHAD againts ground truth methods: reconstruction error (**left-top**), F1-scores (**left-bottom**), and score correlation analysis (**right**).

This separability is further quantified in the bar charts of Figure 4, which highlight a massive disparity in mean reconstruction errors. Specifically, under the 99th Percentile ground truth, the mean error for anomalies (26.871) is orders of magnitude higher than that of normal data (0.057). This sharp signal-to-noise ratio confirms that the model establishes a clear decision boundary between normal and abnormal events. Furthermore, the correlation scatter plot between HIPER-CHAD anomaly scores and reconstruction errors reinforces the efficacy of the hybrid approach. The moderate correlation (r=0.399) suggests that the metrics are complementary rather than redundant. While higher anomaly scores generally align with elevated reconstruction errors (MSE), the presence of data points with moderate MSE but high HIPER-CHAD scores indicates that the inclusion of the KL-divergence term successfully captures probabilistic anomalies such as subtle distribution shifts that simple reconstruction error might overlook.

### 3.2. Time-Step Sensivity Analysis

**Figure 5:**
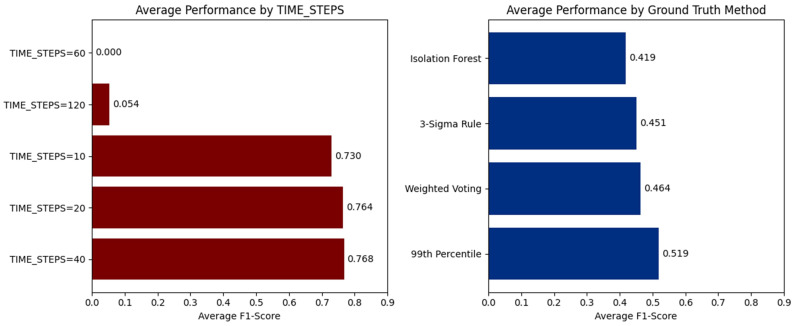
Aggregated performance analysis: Effect of time-step length and ground truth strategy on HIPER-CHAD accuracy.

The selection of time-step window length is pivotal in influencing the sensitivity and stability of the detection model. The Time-Step Sensitivity Analysis is designed to systematically assess the impact of varying the window length parameter on the performance of the model workflow when applied to multivariate time-series sensor data. The analysis reveals a distinct non-linear relationship between the time-step window and performance. Visual evidence from the aggregated F1-scores in Figure 5 demonstrates that HIPER-CHAD consistently achieves its strongest performance within the 10–40 time-step range, where average F1-scores remain high at 0.730, 0.764, and 0.768, respectively. The observed stability in high performance indicates that temporal windows of 100–400 min are sufficient to capture essential temporal dependencies, while avoiding the introduction of extraneous noise. In contrast, performance collapses at longer windows: at 60 time steps, the model fails entirely (F1 = 0.000), and at 120 time steps performance remains negligible (F1 = 0.054), illustrating a catastrophic loss of sensitivity when the sequence length becomes excessively large. Complementary evidence from the right panel of the Figure 5 shows that statistical ground truth definitions, particularly the 99th Percentile (average F1 = 0.519) and Weighted Voting (0.464), yield consistently stronger evaluations than Isolation Forest (0.419), reinforcing that the stability of the model’s performance is tightly coupled with label reliability. Together, these results validate that the optimal temporal window for HIPER-CHAD lies between 10 and 40 time steps, with 20–40 steps offering the most reliable balance between temporal context and detection stability.

**Figure 6:**
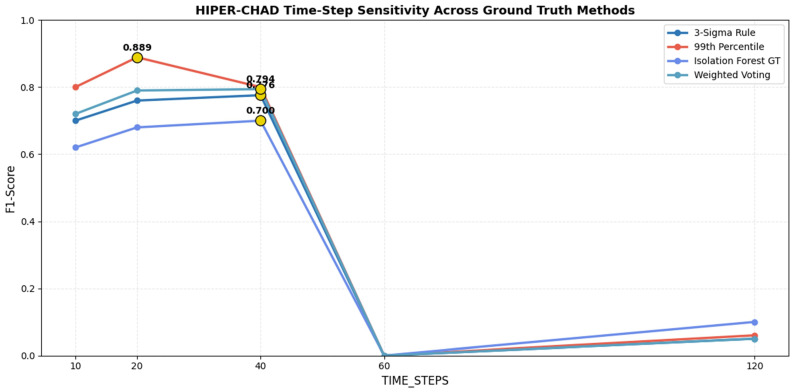
HIPER-CHAD time-step sensivity across ground truth definitions.

This process involves benchmarking HIPER-CHAD detection results across multiple window lengths (e.g., 10, 20, 40, 60, 120 time steps) and comparing F1-score outcomes for each setting against several ground truth labeling strategies (3-Sigma Rule, 99th Percentile, Isolation Forest, Weighted Voting) As illustrated in Figure 6 and Table 2. Line plots further detail the trend of model performance as the time-step window length increases, enabling clear identification of the most effective configuration for capturing anomalous patterns while maintaining both high sensitivity and reliability.

Crucially, the comparative line plots reveal a consistent performance gap beyond 40 time steps. Performance dropped dramatically at 60 time steps, where the F1 score plummeted to near zero on all ground truth methods, and remains negligible at 120 steps. This sharp degradation suggests that overly long sequences introduce excessive noise and dilute the predictive power of the LSTM, likely due to the vanishing gradient problem or the inclusion of irrelevant historical data that obscures recent local dynamics. According to Table 2, A window length of 20 time steps provided the best optimal results for our indoor environment dataset. However, this should not be interpreted as a universally applicable optimal setting. Likewise, when averaged across the four ground-truthing strategies, the 99th Percentile consistently yields the highest mean F1-score, confirming its suitability as the most stable and informative supervisory reference for this dataset. The time-step length in our framework controls the temporal context provided to the LSTM predictor, and thus depends on the characteristic time scales and sampling frequency of the specific application. In other domains, the optimal number of time steps must be determined empirically or guided by domain knowledge.

### 3.3. Comparison Across Multiple Ground Truth Methods

**Figure 7:**
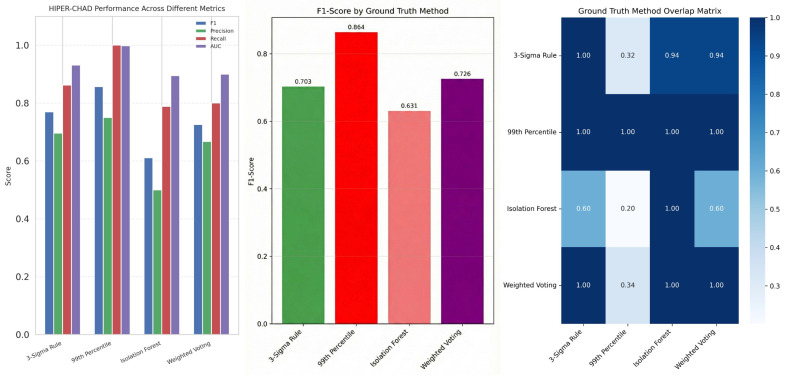
Comparative evaluation of HIPER-CHAD performance and groundt-truth consistency across labeling strategies. HIPER-CHAD performance across multiple evaluation metrics (**left**), F1-score comparison by ground truth definition (**middle**), Ground truth label overlap matrix (**right**).

To thoroughly assess the anomaly detection capabilities of HIPER-CHAD, it is imperative to evaluate the model’s performance across a range of ground truth labeling strategies. Each strategy provides a distinct statistical or algorithmic framework for identifying anomalous events. Figure 7 provides a comprehensive comparison of HIPER-CHAD’s detection metrics across four widely adopted ground truth methods. This comparative analysis enables assessment of the model’s reliability and generalizability independent of the chosen label definition. HIPER-CHAD achieves its highest performance when evaluated against the 99th Percentile method, with an F1-score of 0.857, perfect recall (1.000), and near-perfect ROC-AUC (0.998), signifying extremely effective detection with minimal missed anomalies. The 3-Sigma rule and weighted voting method showed fairly strong performance, though with slightly lower scores, while the Isolation Forest method produced the lowest metrics, possibly due to higher variance and ambiguity in truth assignment.

The comprehensive comparison across different ground truth methods is further reinforced by Figure 7, which expands the evaluation of HIPER-CHAD’s performance in multivariate anomaly detection. It visualizes numerical metrics under each ground truth scenario using bar charts that facilitate direct comparison. The heat map of the ground truth method overlays provides quantitative evidence of label consistency. It shows a high level of agreement (similarity index close to 1.0) between statistical methods such as the 3-Sigma Rule and the 99th Percentile, indicating a strong consensus on what is considered a true anomaly. The overlap matrix yields a 1.0 similarity between the 99th Percentile method and all other ground truth definitions. It might be due to class cardinality based on Figure 2: the 99th percentile produces the fewest number of anomaly labels (36 anomalies), all of which are also detected by the larger anomaly sets from the 3-Sigma Rule (114 anomalies), Isolation Forest (179 anomalies), and Weighted Voting (107 anomalies). Therefore, the overlap is not an artefact, but rather a natural result of a conservative labeling scheme, in which the detected anomalies form a strict subset of other strategies.

**Figure 8:**
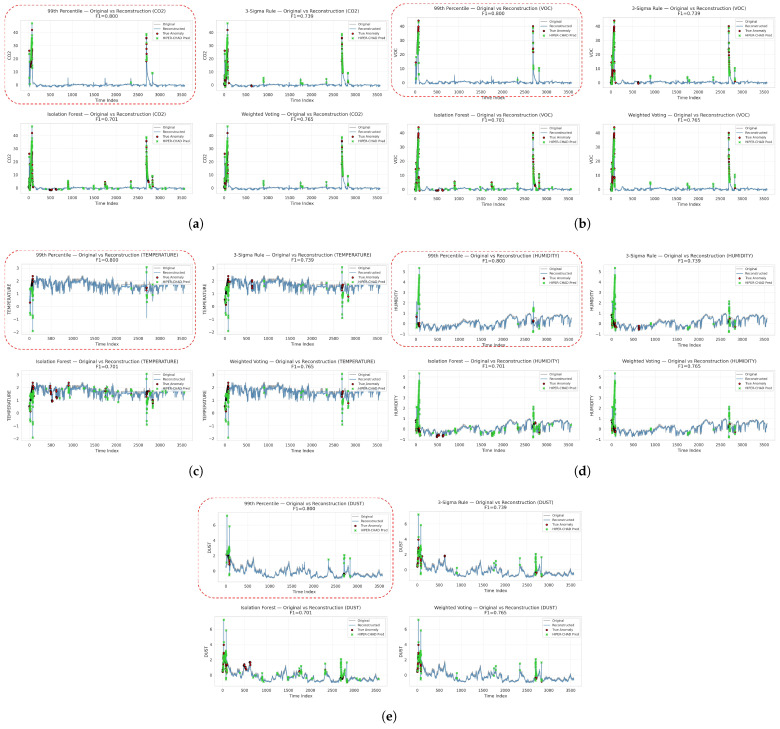
The visualization of original vs reconstruction of indoor enviromental multivariate time-series data and detection consistency across ground truth methods overtime. (**a**) $CO_{2}$ signal overtime. (**b**) VOC signal overtime. (**c**) Temperature signal overtime. (**d**) Humidity signal overtime. (**e**) Dust concentration overtime.

The divergence is clearly illustrated in the comparative signal plots in Figure 8. Based on the definitions of 99th Percentile and 3-Sigma, the ground truth labels coincide with sharp spikes in the $CO_{2}$, VOC, temperature, humidity, and dust signals, and HIPER-CHAD accurately aligns its predictions with these dominant deviations. On the other hand, Isolation Forest produces labels that are scattered across many minor fluctuations, but HIPER-CHAD only responds selectively to physically meaningful peaks, demonstrating its ability to suppress noise-induced detections. This trend remains consistent across all sensor modes: even when temperature and humidity anomalies appear as smaller amplitude deviations than other variables, HIPER-CHAD continues to track the structural shifts present in the reconstructed signal rather than the raw number of outliers. Similarly, although Weighted Voting provides a more balanced reference, HIPER-CHAD still converges closely to the actual anomaly clusters rather than sporadic spikes, indicating that the model’s detection behaviour is more influenced by learned temporal patterns than thresholds triggered by artefacts.

## 4. Discussion

The experimental results present compelling evidence that the HIPER-CHAD model, characterized by its two-stage hybrid architecture, offers a superior approach to multivariate time-series anomaly detection, particularly for analyzing complex indoor environmental data.

### 4.1. Architecture of the Hybrid Approach

The core innovation of HIPER-CHAD lies in the decoupling of temporal modeling from anomaly scoring. By using an LSTM to predict the next state and a VAE to model the distribution of the resulting residual error, the model overcomes key limitations of monolithic deep learning approaches like the standard LSTM Autoencoder. In a conventional Long Short-Term Memory Autoencoder (LSTM-AE), the model is responsible for simultaneously learning both the normal temporal patterns and the standard reconstruction of the data. When an anomaly occurs, the anomaly score—indicated by the reconstruction error—directly measures the model’s inability to accurately reconstruct the anomalous data point. This situation can be problematic, as the autoencoder may inadvertently learn to partially reconstruct common anomaly patterns, resulting in a diluted anomaly score and diminished sensitivity.

In contrast, HIPER-CHAD’s VAE is trained on the residual error distribution of normal predictions. This residual vector serves as a more abstract and focused representation of the system’s unexpected behavior. The VAE’s objective is to learn the compact, low-variance distribution of normal prediction errors. When an anomaly arises, the LSTM predictor becomes ineffective, leading to the creation of a large and structurally unique residual vector. The VAE, having no prior experience with such a residual, struggles to reconstruct it accurately, resulting in a notable reconstruction error. Furthermore, this causes its latent representation to significantly deviate from the standard normal distribution, leading to an increased KL divergence. The integrated score, which combines the reconstruction error with the KL divergence, functions as a highly sensitive and probabilistically valid measure for detecting anomalies. Sensitivity analysis of the KL divergence weight parameter (β) shows that removing the probabilistic term (β=0) results in a significant decrease in performance (an F1-score decrease of around 8–10%), confirming the importance of the VAE latent distribution constraint in the HIPER-CHAD architecture.

These architectural decisions are supported by the empirical outcomes: HIPER-CHAD attains an F1-Score of 0.8571, surpassing both the leading temporal deep learning model (LSTM-AE, F1 = 0.8095) and the best-performing standard VAE (F1 = 0.7816). The perfect recall rate of 1.0000 achieved by HIPER-CHAD further highlights the effectiveness of this residual-based approach in accurately capturing all true anomalies without overfitting to noise.

### 4.2. The Critical Role of the Time-Step Window

The time-step sensitivity analysis, as shown in Table 2 and Figure 6, provides crucial practical insights. The determination of the lookback window is a pivotal factor that significantly influences performance metrics. The optimal window, identified as 20 time steps (equivalent to 200 min), demonstrates that typical temporal dependencies in indoor environments such as the natural fluctuations of ${\mathrm{CO}}_{2}$ and temperature are most accurately captured within a timeframe of approximately three to four hours.

A significant reduction in performance is observed as window sizes extend beyond this optimal range (e.g., 60, 80, 120 time steps). This degradation can be explained by two main factors. Firstly, longer sequences tend to introduce increased levels of non-stationarity and accumulated noise, which complicates the prediction task for the LSTM model. Secondly, data from the distant past becomes less relevant for predicting near-future events, leading to a “dilution” of the most recent and pertinent temporal features. This finding emphasizes the importance of precise hyperparameter tuning in time-series anomaly detection and argues against the simplistic approach of maximizing sequence length. Specifically, in the domain of indoor environmental monitoring, a lookback window of 3–4 h appears optimal for capturing predictive patterns effectively.

While a window length of 20 time steps yields the best performance for the indoor environmental dataset considered in this study, this setting should not be interpreted as universally optimal. The temporal dynamics, disturbance patterns, and sampling frequencies in outdoor or other external monitoring scenarios can differ substantially. Therefore, when HIPER-CHAD is applied to other types of multivariate sensor data, the time-window length and related hyperparameters must be recalibrated and validated on representative domain-specific datasets.

### 4.3. Comparative Analysis with State-of-the-Art

The comparison against eight other models provides a robust benchmark. The poor performance of Local Outlier Factor (LOF, F1 = 0.0741) highlights the inadequacy of purely distance-based, non-temporal methods for this complex, high-dimensional time-series data. Traditional machine learning models, such as Isolation Forest (F1 = 0.7532) and One-Class SVM (F1 = 0.5496), demonstrate moderate success. However, their inability to explicitly model temporal dependencies constrains their overall performance when compared to deep learning models.

In the evaluation of deep learning models, the LSTM Autoencoder achieved the highest performance among baselines, with an F1 score of 0.8095. This result emphasizes the pivotal role of recurrent layers in effectively handling temporal data. However, the superior results of HIPER-CHAD highlight the distinct advantages of the VAE-on-residuals methodology. The Standard VAE (F1 = 0.7816) also demonstrated reasonable performance due to its ability to capture compact probabilistic representations, but it was limited by the absence of an explicit temporal model (trained on flattened sequences). Conversely, TCN Autoencoder and Standard Autoencoder showed lower effectiveness (F1 of 0.6476 and 0.6207, respectively), indicating that for this dataset, the LSTM’s ability to retain long-term memory is more advantageous than the local feature extraction of TCNs or the non-temporal characteristics of standard AEs.

It is important to note that this experimental evaluation relies on an indoor environmental monitoring dataset, where temporal dynamics and disturbance patterns (e.g., occupancy, HVAC operation) are relatively constrained. Consequently, the optimal window length and specific performance metrics are context-dependent. Nevertheless, the core principles of HIPER-CHAD—separating temporal prediction from residual modeling and utilizing a residual-based VAE—are generalized and can be widely applied to multivariate sensor data. For outdoor or volatile environments, hyperparameters (e.g., time window length, network capacity, threshold) would require recalibration.

## 5. Conclusions

This study presents a comprehensive evaluation of the Hybrid Integrated Prediction-Error Reconstruction-based Anomaly Detection (HIPER-CHAD) model, specifically designed for multivariate indoor environmental time-series data. The innovative two-stage architecture, which combines an LSTM predictor with a Variational Autoencoder trained on residual errors, has demonstrated robust performance. The key findings are summarized as follows:

- HIPER-CHAD achieved the highest F1-Score of 0.8571 against the 99th Percentile ground truth, demonstrating a distinct performance advantage over all eight comparative baseline models and three ground truth methods, including hybrid deep learning architectures such as the LSTM Autoencoder (F1=0.8095). Notably, the model achieved a perfect recall (1.0000), indicating its reliability in identifying all true anomalies without excessive false negatives.
- The results validate the core hypothesis that modeling the probability distribution of prediction residuals is a more effective strategy than modeling raw data reconstruction. The integrated anomaly score—combining reconstruction error magnitude with KL divergence—creates a high degree of class separability, effectively distinguishing genuine system deviations from stochastic noise.
- Sensitivity analysis revealed that the model’s stability is highly dependent on the temporal context. A lookback window of 20 time steps (approximately 200 min) was identified as the optimal configuration for indoor environmental monitoring, balancing the need to capture thermal/gas inertia trends while minimizing the noise introduced by excessively long sequences.

The HIPER-CHAD model offers a reliable, interpretable, and mathematically grounded framework for anomaly dete;ction in multivariate time series. These findings strongly support its viability for real-time monitoring in critical infrastructures, such as smart buildings and industrial IoT systems, where timely and precise detection is essential. While this study is limited to indoor environmental data, future work will explore the model’s adaptability to the higher volatility of outdoor environments and other domains (e.g., industrial sensor networks). Further research will also focus on integrating unsupervised learning mechanisms and exploring alternative generative models to further refine the residual error distribution learning.

## Figures and Tables

**Table 1:** Performance Summary of Anomaly Detection Models Against Ground Truth.

Model Name	Category	F1-Score	Precision	Recall	ROC-AUC
Proposed Model (HIPER-CHAD)	Hybrid	0.8571	0.7500	1.0000	0.9983
LSTM Autoencoder [26]	Hybrid	0.8095	0.7083	0.9444	0.9702
VAE [10]	Deep Learning	0.7816	0.6667	0.9444	0.9698
Isolation Forest [34]	Traditional ML	0.7532	0.7073	0.8056	0.9011
TCN Autoencoder [35]	Hybrid	0.6476	0.4928	0.9444	0.9673
Autoencoder (AE) [36]	Deep Learning	0.6207	0.4500	1.0000	0.9938
PCA Reconstruction [37]	Traditional ML	0.5669	0.3956	1.0000	0.9922
One-Class SVM [34]	Traditional ML	0.5496	0.3789	1.0000	0.9917
Local Outlier Factor [38]	Traditional ML	0.0741	0.0505	0.1389	0.5561

**Table 2:** HIPER-CHAD Performance vs. Time-Step Window Size.

Time Steps	Average F1-Score	Optimal F1-Score (99th-Percentile)
10	0.737	0.770
20	0.839	0.884
40	0.149	0.150
80	0.089	0.090
120	0.091	0.092

## Data Availability

The dataset used in this study is publicly available and can be accessed through the following repository link: https://raw.githubusercontent.com/vandhapw/datasets/refs/heads/main/filtered_data.csv (accessed on 10 October 2025).
